# The Role of Autopsy in the Forensic and Clinical Evaluation of Head Trauma and Traumatic Brain Injury in Road Traffic Accidents: A Review of the Literature

**DOI:** 10.3390/diagnostics15040442

**Published:** 2025-02-12

**Authors:** Matteo Antonio Sacco, Maria Cristina Verrina, Roberto Raffaele, Saverio Gualtieri, Alessandro Pasquale Tarallo, Santo Gratteri, Isabella Aquila

**Affiliations:** Institute of Legal Medicine, Department of Medical and Surgical Sciences, “Magna Graecia” University of Catanzaro, 88100 Catanzaro, Italy; matteoantoniosacco@gmail.com (M.A.S.); mariacristina.verrina@studenti.unicz.it (M.C.V.); raro81@libero.it (R.R.); saverio.gualtieri@studenti.unicz.it (S.G.); alessandropasquale.tarallo@studenti.unicz.it (A.P.T.); gratteri@unicz.it (S.G.)

**Keywords:** head injury, road traffic accidents, autopsy

## Abstract

Road traffic accidents (RTAs) are a leading cause of morbidity and mortality worldwide, frequently resulting in traumatic brain injuries (TBIs), skull fractures, and spinal injuries. This manuscript examines the forensic aspects of head trauma caused by RTAs, focusing on the role of autopsy and imaging in diagnosing and characterizing injuries. Through a systematic review of the literature, the study highlights the mechanisms of injury, including high-speed collisions, whiplash, and pedestrian impacts, and explores their pathological consequences, such as subarachnoid hemorrhage, intracranial hemorrhage, and diffuse axonal injury. The differentiation between traumatic and non-traumatic conditions, such as aneurysmal subarachnoid hemorrhage, is emphasized to ensure accurate clinical and forensic assessments. Advances in imaging technologies, particularly virtopsy, are discussed for their potential in non-invasive documentation and analysis of head injuries, while limitations of this approach are acknowledged. Furthermore, the manuscript underscores the importance of preventive measures, including helmet and seatbelt use, vehicle safety innovations, and improved road design, in reducing the incidence and severity of RTAs. By integrating clinical, forensic, and preventive perspectives, this study provides a comprehensive framework for understanding and addressing the burden of head trauma related to RTAs.

## 1. Introduction

Road traffic accidents (RTAs) are a major cause of death and disability [1]. In 1990, injuries, overall, were ranked as the 12th largest contributor to the global disease burden in terms of disability-adjusted life years (DALYs), rising to 7th place in 2010, and projected to reach 5th by 2030. Road accidents account for approximately 30% of injury-related deaths worldwide. Globally, road accidents are the leading cause of death among young people aged 9 to 25 years, and more people die from road accidents than from HIV/AIDS, tuberculosis, or diarrheal diseases [1,2]. A recent report from the World Bank and the World Health Organization indicated that injuries would be among the major global health problems in this millennium.

Road traffic accidents (RTAs) represent the most common injury mechanism in many countries. In its report, the WHO estimated that 1.2 million people die in road accidents every year, with many more people injured and disabled. These figures are expected to rise dramatically if no action is taken, particularly in the field of prevention. It is important to state that RTA deaths occur in the lowest age groups; in the vast majority of cases, the victims are under 50 years old. Disability-adjusted life years (a composite measure of time lost due to premature death and time spent with a disability) show that RTAs are a major hazard to society, with a significant effect on the workforce, as many of those injured are young. The burden on health services is enormous.

In Uganda, a study at Mulago Hospital in Kampala found that road accidents accounted for 35% of all injuries, with 697 patients in the 11 weeks between 25 September and 11 December 1997. In common with many reports from developing countries, pedestrians were the largest group of patients, followed by vehicle occupants and then cyclists. Of the 25 children under the age of 10 injured, 17 were injured while on foot. In Cape Town, child pedestrians involved in motor vehicle accidents are the most common source of serious pediatric head injuries. This was the mechanism of injury in 53 out of 102 hospitalized children. Data on the impact of RTAs are now available from around the world [3,4,5,6].

Every year, more than 10 million people are affected by traumatic brain injury (TBI). Despite efforts to improve care for TBIs, they remain a public health problem, continuing to cause high mortality and morbidity in a young population. The World Health Organization (WHO) considers TBI to be one of the most urgent and under-recognized areas of public health problems. The primary causes of traumatic brain injury (TBI) vary globally. In low- and middle-income countries (LMICs), road traffic accidents (RTAs) contribute significantly to the TBI burden, whereas in high-income countries, falls—particularly among the elderly—are the leading cause [7]. Road accidents account for 2.5% of total deaths worldwide, and the WHO predicts that by 2030, RTAs will become the seventh leading cause of death, increasing from 2.5% in 2015 to 2.6% in 2030 of total deaths in the world [4]. This morbidity and mortality are largely related to the frequency and severity of brain injuries caused by this type of accident. Head trauma is considered the main cause of death following road accidents. On the other hand, most epidemiological studies on head injuries are not exclusively dedicated to head injuries related to road accidents, as they often also include other mechanisms (falls, assaults, etc.) [8]. The rate of TBI resulting from traffic accidents is highest in Africa and Southeast Asia (both 56%), and lowest in North America (25%) [5].

Traumatic brain injuries (TBIs) are among the most severe injuries, with their leading causes varying by region and socioeconomic context, including road traffic accidents, falls, and other traumatic events [6,7,8,9]. Traumatic brain injuries (TBIs) constitute a significant proportion of head injuries resulting from road traffic accidents. A staggering number of TBIs from motor vehicle crashes lead to emergency department visits, hospitalizations, and a considerable number of deaths annually. These injuries are especially prevalent among males aged 15 to 35 years, who are more frequently involved in such accidents. TBIs can result in long-term cognitive, physical, and emotional impairments, often necessitating prolonged medical treatment and rehabilitation.

The aim of this review is to analyze, from a forensic point of view, head trauma resulting from road accidents, comparing the different injuries produced during these traumatic events. This study emphasizes the role of the autopsy as a vital examination for the study, description, and characterization of these lesions for forensic purposes. By reading this article, researchers and practitioners will gain a comprehensive understanding of the forensic and clinical aspects of head trauma, enabling them to improve diagnostic accuracy, reconstruct accident dynamics, and inform preventive strategies aimed at mitigating the impact of RTAs.

## 2. Materials and Methods

A narrative review of the literature was carried out using academic web browsers such as PubMed, NCBI, and Scopus, to identify articles describing head trauma resulting from road accidents. Keywords used for the search included “head trauma”, “forensic”, and “road traffic accidents”. The review included all types of works investigating the characteristics of injuries caused by road accidents, including original articles, case reports, and retrospective and prospective case series.

To account for the diversity in objectives, methodologies, and definitions across the included studies, a clear categorization was established. Clinical and non-clinical sources were reviewed separately. Clinical publications were further examined for variability in the definition, methodology, and treatment of patients with traumatic brain injuries (TBIs). The inclusion criteria focused on studies that explicitly described the mechanisms of injury, severity assessment, and treatment outcomes. Articles that lacked clear descriptions or did not meet these criteria were excluded. This distinction ensured that only studies meeting the required standards were included in the analysis.

The following types of studies were included: forensic studies involving autopsies of victims to analyze injury distribution and mechanisms, clinical studies providing detailed information on living and deceased patients, and public health and traffic engineering studies addressing prevention strategies and technologies. Titles, abstracts, and full texts were screened, and the references of selected articles were cross-referenced to identify additional relevant studies.

To provide clarity and uniformity, the key terms used in this review are defined as follows:

- Head trauma: Injury to the scalp, skull, or brain caused by external mechanical forces.
- Severity of trauma: Classified using the Glasgow Coma Scale (GCS), with scores ranging from mild (13–15), to moderate (9–12), to severe (≤8).
- Concussion: A mild traumatic brain injury characterized by transient alteration in brain function without structural damage.
- Focal TBI: Localized brain injuries such as contusions or hematomas, often visible on imaging.
- Diffuse TBI: Widespread damage resulting from shearing forces, frequently associated with diffuse axonal injury.
- Spine injury: Damage to the vertebral column or spinal cord, often co-occurring with severe road traffic accidents.
- Virtopsy: Non-invasive imaging techniques, such as CT or MRI, used in forensic investigations to assess injuries without traditional dissection.

## 3. Results and Discussion

### 3.1. The Role of the Forensic Pathologist and Autopsy Investigations

Traumatic injuries from road accidents that can affect the head are heterogeneous [10,11,12,13,14]. Typically, the areas involved in these lesions are the skull, the brain, and the cervical spine. The role of autopsy by a forensic pathologist is revealed to be crucial in the differential diagnosis of these injuries, in assessing the degree of severity of the injuries, and in providing useful elements for the reconstruction of the dynamics of the accident.

The analysis during the autopsy requires an accurate evaluation, firstly during the head’s external examination. It is important that each injury (bruises, ecchymoses, lacerated or lacerated-contused wounds) is correctly described and measured. Furthermore, the pathologist will proceed with an evaluation of any fractured areas already noticeable upon palpation. Once the scalp tissues have been detached, an accurate analysis of the galea capitis is essential, paying attention to hemorrhagic infiltration areas. We suggest that these areas be incised and taken for histopathological investigations to discriminate their vitality. The analysis of the skullcap is fundamental, particularly regarding the identification of the impact areas or any blows or recoils. For this purpose, the temporalis muscles must be cut, and it is necessary to investigate any infiltration areas.

After opening the skullcap, it is essential to carry out a gross analysis of the brain, with evaluation of the cerebral hemispheres and subsequent fresh analysis. The pathologist can thus identify, through various sections (such as the Virchow cut), areas of subarachnoid or intraparenchymal hemorrhages, areas of hemoventricle, or traumatic lesions to the brainstem. The differentiation between traumatic and non-traumatic conditions, such as traumatic subarachnoid hemorrhage (TSAH) and aneurysmal subarachnoid hemorrhage, is essential during this phase in order to avoid misinterpretations. TSAH can occur in all degrees of traumatic brain injury (TBI) severity, but has variable clinical implications depending on the extent of the hemorrhage [15,16]. The analysis also emphasizes the extent of the lesions (size and depth), as well as the need to carefully evaluate the brainstem using specific sections. Subsequently, the cranial cavities must be visualized, and any fractures of the base or vault of the skull must be measured and photographically documented.

Autopsies in cases of cranial trauma resulting from road traffic accidents often present unique challenges, which require a combination of meticulous examination and advanced techniques to resolve. One of the primary difficulties is the identification of subtle or hidden injuries that may not be immediately apparent during gross examination. For instance, diffuse axonal injury (DAI), a common consequence of high-velocity impacts, is typically microscopic, and requires specialized staining techniques, such as β-amyloid precursor protein (β-APP) immunohistochemistry, to confirm. Another challenge involves distinguishing between primary and secondary injuries, particularly in complex cases involving multiple impact points. This distinction is critical for reconstructing the sequence of trauma and determining the cause of death.

Forensic pathologists must also address the difficulty of correlating external injuries with internal damage. Fractures of the cranial base, for example, may be challenging to detect without thorough dissection, and may be associated with dural tears or cerebrospinal fluid (CSF) leaks. Advanced imaging, such as postmortem CT (PMCT), can complement the autopsy by guiding the dissection to areas of interest, but definitive diagnosis often requires direct anatomical examination.

Additionally, the evaluation of rotational and deceleration injuries, such as those sustained in rear-end collisions, poses significant challenges. These injuries often produce shearing forces that result in microvascular disruptions and brainstem trauma, which are difficult to detect without detailed histopathological analysis. Pathologists also face difficulties in assessing the extent of secondary injuries, such as those caused by increased intracranial pressure (ICP) or systemic complications like fat embolism syndrome (FES).

### 3.2. Traumatic Brain Injury

High-speed collisions are a significant cause of head injuries in road traffic accidents (RTAs) [9]. When vehicles travel at high speeds, the force of impact during a collision can lead to severe trauma, including traumatic brain injuries (TBIs). Concussions are among the most prevalent types of head injuries sustained during RTAs. This type of injury often occurs when the brain shifts forcibly within the skull, either due to impact or rapid changes in momentum, such as in a sudden stop or collision. However, not all concussions involve loss of consciousness, as this symptom depends on the dynamics and severity of the trauma. Consulting with clinicians such as neurologists or neurosurgeons is essential to ensure accurate differentiation between these terms and their implications [11].

Pedestrians involved in traffic accidents frequently suffer from concussions, making them one of the most common injuries among this group. The pedestrian’s head often impacts the vehicle during the overtaking phase, or the roof of the car during vaulting. In other cases, the pedestrian may be thrown, leading to secondary head impacts on the ground. The symptoms of concussions can range from mild to severe, involving headaches, dizziness, confusion, and sometimes loss of consciousness.

When a vehicle collides at high speed, the occupants’ heads can strike hard surfaces within the car, such as the dashboard or windshield. The impact force causes the brain to crash against the skull’s interior, leading to bruising, bleeding, and shearing of brain tissues [12]. Such collisions can cause multiple types of brain injuries, including subarachnoid hemorrhage (SAH) and subdural hematoma, which are common in these scenarios [12]. It is important to distinguish traumatic SAH, caused by mechanical forces during an accident, from aneurysmal SAH, which results from arterial rupture due to pre-existing vascular conditions. These two conditions represent distinct pathological entities, and must be differentiated to avoid diagnostic errors [15]. Traumatic SAH can occur in all degrees of TBI severity, and its consequences vary depending on the extent and location of the hemorrhage [16].

The brain’s movement inside the skull during these high-impact events can be devastating, often resulting in catastrophic outcomes. Whiplash and sudden deceleration in RTAs play a crucial role in causing cerebral hemorrhage. Whiplash occurs when the head is abruptly jerked back and forth, commonly during rear-end collisions, creating a rapid acceleration–deceleration motion [13]. The sudden deceleration forces experienced by vehicle occupants during crashes can lead to significant head trauma, as the brain is subjected to intense inertial forces [13]. When a high-impact collision occurs, the force can rupture blood vessels within the brain, leading to intracerebral hemorrhage (ICH), which can cause significant brain damage due to the accumulation of blood and the subsequent increase in intracranial pressure.

Moreover, secondary injury mechanisms, such as heme toxicity, exacerbate the damage by triggering cellular pathways that lead to neuronal death and inflammation [13]. Subarachnoid hemorrhage (SAH) is another critical condition frequently linked to RTAs. Bleeding into the subarachnoid space typically occurs due to rupture of an arterial vessel caused by high-impact trauma [15]. Traumatic subarachnoid hemorrhage (TSAH) is commonly observed in RTA victims, and is associated with severe outcomes, including death and significant neurological impairment [16]. TSAH is frequently observed following TBI, and has been associated with secondary injury mechanisms. However, traumatic intracerebral hemorrhages, including cerebral contusions, can undergo secondary expansion in more than 60% of cases, even in the absence of TSAH. The exact relationship between TSAH and contusion progression remains a subject of investigation. Some studies suggest that TSAH may contribute to altered cerebral perfusion dynamics, exacerbating contusion expansion in certain cases [15]. Further research is needed to elucidate this relationship [16]. Motor vehicle collisions (MVCs) are strongly associated with these injuries. Furthermore, patients with TSAH exhibit a higher mortality rate, with MVCs linked to a three-fold increased risk of mortality [17].

This correlation underscores the critical need for enhanced safety measures and vehicle design improvements to mitigate the impact of collisions. Hemorrhage is diagnosed in more than 50% of all RTA victims with severe traumatic brain injury (sTBI), ranging from 44.9% in motorists to 63.6% in pedestrians [18]. Pre-existing medical conditions, such as hypertension or clotting disorders, can exacerbate the effects of trauma sustained in RTAs, increasing the likelihood of cerebral hemorrhage. Hypertension weakens vascular walls, making them more prone to rupture, while clotting disorders impair the body’s ability to control bleeding effectively. This combination significantly complicates recovery and prognosis [10].

The clinical presentation of cerebral hemorrhage and subarachnoid hemorrhage (SAH) following RTAs often includes sudden and severe headache, which is the hallmark symptom of SAH [19]. Other common symptoms include nausea, vomiting, lethargy, and seizures. These initial presentations necessitate a rapid and thorough assessment to determine the extent and nature of the brain injury. Clinicians must be vigilant in recognizing these symptoms, as they can progress quickly and result in significant neurological damage if not promptly addressed.

**Figure 1:**
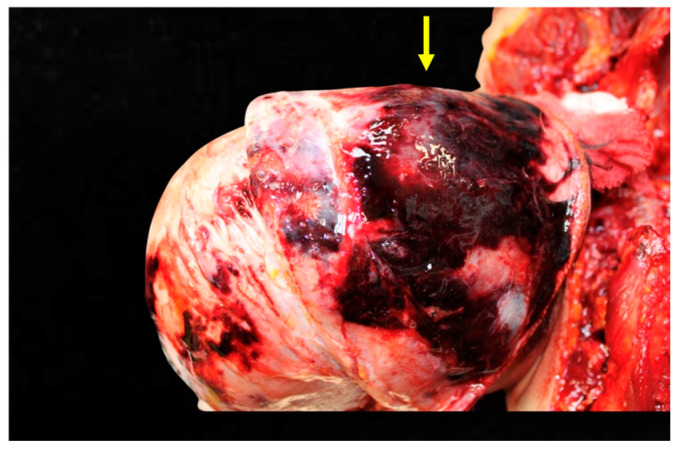
Analysis of head trauma in the frontal region. This image depicts significant bruising and lacerations on the frontal area of the skull. The external injuries suggest a high-energy impact consistent with head-on collisions.

**Figure 2:**
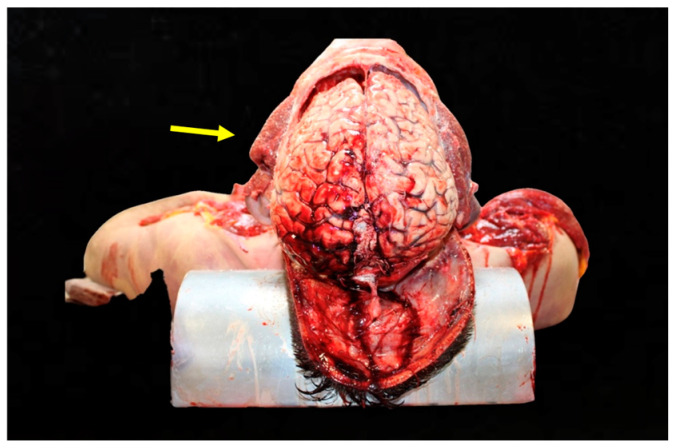
Cerebral hemorrhage found upon opening of the skull. This figure reveals extensive subarachnoid hemorrhage (SAH) visible upon gross examination. The bleeding pattern suggests rupture of vessels in the subarachnoid space, likely due to rapid deceleration forces during the accident.

**Figure 3:**
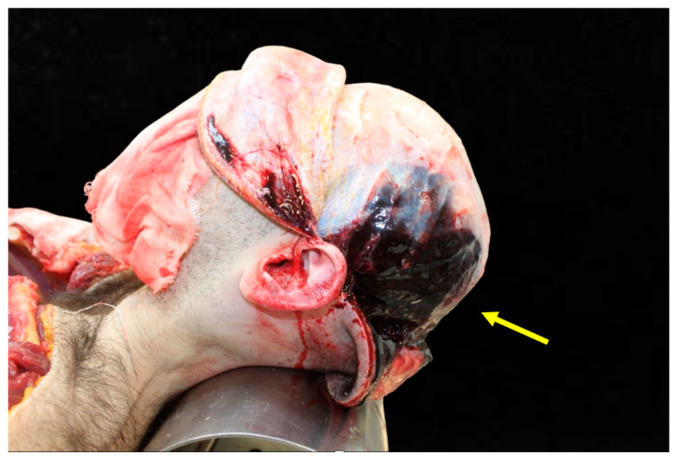
Trauma of the left parietotemporal region. The illustration shows localized contusions and fractures in the left parietotemporal region, correlating with lateral impacts.

**Figure 4:**
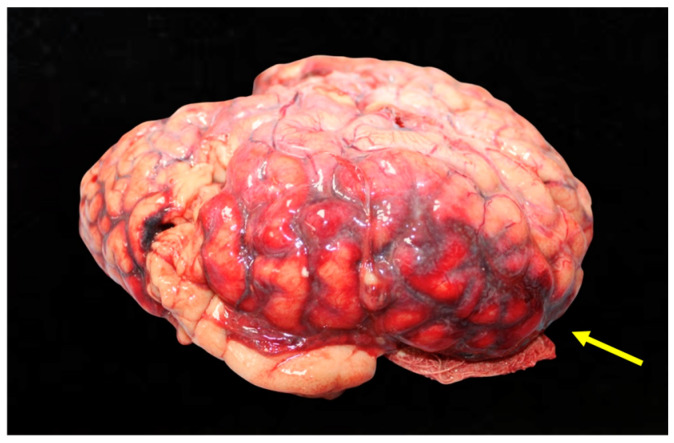
Detailed view of the bleeding into the subarachnoid space, with clear demarcation of the hemorrhage.

**Figure 5:**
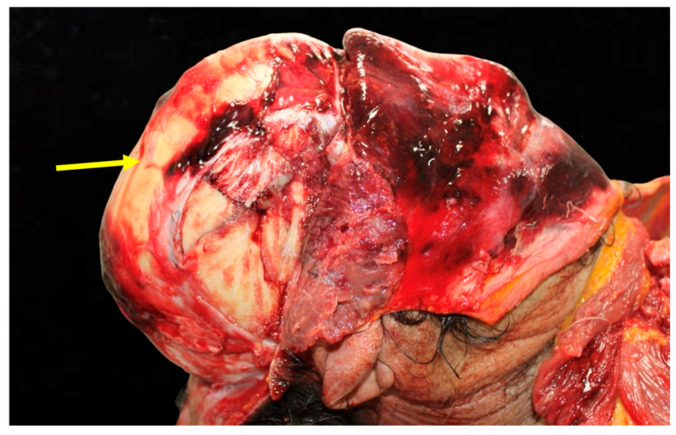
Hemorrhagic infiltration extending into the temporalis muscle, which often indicates a high-impact injury. The presence of hemorrhage in soft tissues supports findings of significant blunt force trauma.

**Figure 6:**
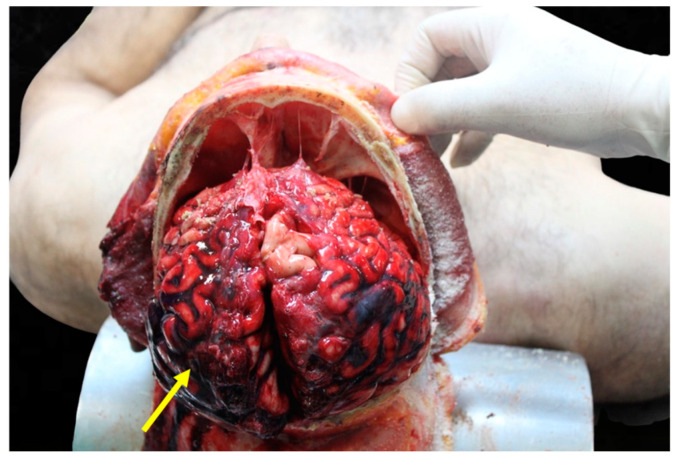
Subdural hemorrhage at skull opening, with extensive bleeding visible on the brain.

Imaging techniques play a crucial role in the diagnosis and management of intracranial hemorrhages in the context of RTAs. Noncontrast CT scans are the preferred modality in acute settings, due to their ability to quickly and accurately identify intracranial hemorrhage [20]. CT is a fast and accurate technique used for the detection of intracranial hemorrhage, mass effect, and edema [21]. However, in cases of subacute and chronic traumatic brain injury (TBI), MRI is superior in identifying parenchymal atrophy and white matter injury [21,22,23]. The choice of imaging technique depends on the timing and specific clinical scenario, with CT being more appropriate for acute assessments and MRI providing detailed evaluations in subacute and chronic phases. (Figure 1, Figure 2, Figure 3, Figure 4, Figure 5 and Figure 6).

### 3.3. Skull Fractures

Skull fractures are another severe consequence of head injuries in RTAs. These fractures often result in more complications and have a higher fatality rate compared to head injuries without fractures [24]. A study examining the frequency of skull base fractures in fatal head trauma cases found that 66.9% of such incidents were attributed to forensic incidents involving road accidents [25]. Skull fractures can lead to additional complications, such as intracranial hemorrhages and infections, which exacerbate the severity of the injury. The presence of a fracture can also indicate a higher level of force applied during the accident, correlating with more severe brain injuries [26].

Skull fractures are generally categorized into several types: linear, depressed, diastatic, and basilar. Linear skull fractures are the most common, and involve a break in the bone that resembles a thin line without splintering, depression, or distortion of bone. Depressed skull fractures, on the other hand, occur when a part of the skull is sunken in due to a direct blow, and these are often more severe because they may compress brain tissue. Diastatic fractures typically occur along the sutures of the skull, widening these natural bone divisions, and are more commonly seen in young children whose sutures have not yet fully ossified. Basilar fractures, which involve the base of the skull, are particularly serious due to their proximity to critical structures. Basilar fractures can directly affect the brainstem, as bone fragments may compress or disrupt this vital structure, leading to severe autonomic dysfunction, including impaired respiratory and cardiovascular control. Furthermore, these fractures often breach the dura mater, causing cerebrospinal fluid (CSF) leaks, which significantly increase the risk of infections such as meningitis.

The severity of these fractures is often indicative of the trauma’s intensity and potential complications, such as intracranial hemorrhage or neurological deficits [27]. Certain impact points on the skull are more prone to fractures due to the structure and vulnerability of the cranial bones. The most common points of impact that lead to fractures include the frontal bone, temporal bone, and occipital bone. The frontal bone, which forms the forehead, is often involved in head-on collisions, and this can result in linear or depressed fractures. The temporal bone, located at the sides of the skull, is particularly susceptible to fractures due to its relative thinness and the presence of crucial structures such as the middle meningeal artery. When ruptured, this artery can lead to epidural hematomas, a life-threatening condition that requires immediate surgical intervention. The occipital bone, at the back of the skull, can be fractured in rear-end collisions or falls, often leading to basilar fractures, which pose significant risks due to their proximity to the brainstem and spinal cord [28,29].

Skull base fractures result from significant blunt force trauma, and often indicate a high-energy impact. The propagation of force through the cranial base can lead to indirect brainstem involvement, particularly when fractures occur near the clivus or petrous portion of the temporal bone. If the impact transmits sufficient force to impair brainstem integrity, this may contribute to trauma-induced unconsciousness. Therefore, the presence of a skull base fracture, particularly in proximity to the brainstem, warrants careful clinical evaluation for associated brainstem injury.

Skull fractures can directly cause brain injuries or serve as a marker for underlying brain trauma. For instance, a depressed skull fracture may push bone fragments into brain tissue, causing contusions or lacerations. Additionally, the presence of a skull fracture significantly increases the likelihood of intracranial hemorrhage, which can be life-threatening. Subdural and subarachnoid hemorrhages are frequently associated with skull fractures [27]. Subdural hemorrhage occurs when bridging veins between the brain’s surface and the dura mater are torn, while subarachnoid hemorrhage results from damage to arteries within the subarachnoid space. These hemorrhages exacerbate intracranial pressure, leading to secondary brain injuries and worsening neurological outcomes. Acute subdural hematomas (aSDHs) typically result from high-impact trauma leading to cerebral contusions with associated hemorrhage. In contrast, chronic subdural hematomas (cSDHs) often originate from slow venous bleeding, primarily from torn bridging veins, and are more common in elderly individuals or those with coagulopathies. The two entities differ in their clinical course, management, and prognosis. aSDHs often require urgent surgical intervention due to rapid neurological deterioration, whereas cSDHs may develop insidiously over weeks, and can sometimes be managed conservatively or with minimally invasive techniques.

**Figure 7:**
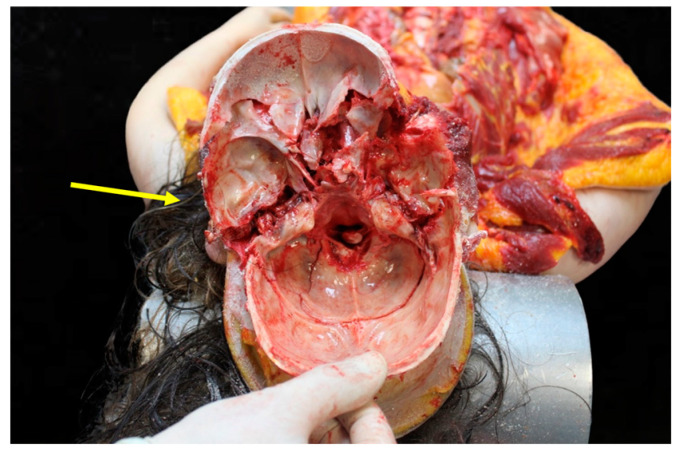
Complete skull base fracture and its extent. The fracture originates from the left middle cranial fossa, traverses the sella turcica, and extends to the anterior cranial fossa. This trajectory crosses critical anatomical structures, potentially compromising the brainstem and cranial nerves, highlighting the severity and life-threatening implications of such injuries.

The combination of skull fractures and intracranial hemorrhages often complicates the clinical picture and necessitates prompt surgical intervention. For example, a basilar fracture accompanied by subarachnoid hemorrhage may require decompressive surgery to alleviate intracranial pressure and repair vascular or dural injuries. Delays in intervention can lead to brain herniation, permanent neurological deficits, or death. Furthermore, the severity of the fracture can correlate with the extent of brain injury, influencing the prognosis and potential for long-term disability. Therefore, the assessment of skull fractures is a critical component in the management of traumatic brain injuries following road traffic accidents (Figure 7).

### 3.4. Spine Injuries

Statistical analysis reveals a concerning number of cervical spine injury cases resulting from road traffic accidents. Approximately 1.77% of all cases of cervical spine injuries result in mortality, often accompanied by chest and abdomen injuries [30]. Moreover, it is estimated that around 869,000 traffic crash-related cervical spine injuries are treated in hospitals annually in the United States [31]. Age and gender distribution studies indicate significant disparities among individuals affected by cervical spine injuries due to RTAs. The majority of victims are male, accounting for 88% of cases, with a median age of 25 years [22]. In female victims, the median age is higher, at 32 years [32]. Additionally, certain age groups are at higher risk, particularly individuals between 20 and 44 years, comprising 68.5% of the affected population [33]. Such demographic data are crucial for developing age- and gender-specific interventions to reduce the incidence of these injuries. Rear-end collisions are particularly associated with the highest relative risk of whiplash injury compared to lateral impacts [34]. These types of collisions often involve sudden deceleration, forcefully snapping the head backward and then forward, which can result in significant neck strain and injury. Additionally, the impact location—whether it is a rear-end or side collision—affects different parts of the neck. For example, rear-end collisions are frequently linked with cervical spondylolisthesis, a condition where one of the cervical vertebrae slips out of place onto the vertebra below it. On the other hand, frontal impacts also pose a risk, but are less likely to result in cervical spine injuries compared to rear-end impacts. Rear-end collisions have been found to cause cervical spine injuries in a ΔV range (change in velocity) between 9 km/h and 37 km/h, highlighting the role of speed in injury severity [35]. Deceleration dynamics, which involve a rapid reduction in vehicle speed, play a significant role in the mechanisms of these injuries. The examination of the entire spine by pathologists involves a combination of imaging techniques and direct anatomical evaluation during the autopsy. Advanced imaging methods, such as computed tomography (CT) and magnetic resonance imaging (MRI), are often employed prior to dissection to identify fractures, dislocations, spinal cord compression, or soft tissue injuries. These imaging techniques provide a comprehensive, non-invasive view of the spinal column and help to target areas of interest for detailed examination.

During the autopsy, the spine is typically examined using a posterior approach [36]. A midline incision is made along the back, and the overlying skin, subcutaneous tissues, and muscles are carefully removed to expose the vertebral column. The vertebrae are then opened using a laminectomy procedure, which involves the removal of the bony posterior elements to access the spinal cord and surrounding structures. This approach allows for a thorough inspection of the spinal cord, dura mater, and vertebral bodies, enabling the identification of hemorrhages, contusions, lacerations, or fractures.

### 3.5. Histopathological Investigations in Head Trauma

Traumatic brain injuries (TBIs) exhibit several common histopathological features. Diffuse axonal injury (DAI) is one of the most frequently observed and critical pathological features in TBI [37]. DAI is characterized by widespread damage to the brain’s white matter tracts, which can disrupt communication between different brain regions. Histologically, DAI presents with axonal swelling, bulb formation, and secondary axotomy. Another common feature is the presence of hemorrhages, which can be classified into epidural, subdural, subarachnoid, and intracerebral types based on their location [38]. Vascular injury and necrotic neuronal death are often observed in severe cases, indicating substantial disruption to the brain’s blood supply and cellular architecture.

While brain edema and breakdown of the blood–brain barrier are prominent features associated with TBI, it is important to clarify that increased intracranial pressure (ICP) cannot be directly determined by histopathological means. Instead, histopathological findings such as vascular congestion, diffuse brain swelling, and evidence of herniation (e.g., uncal or tonsillar) are indicative of the effects of elevated ICP. These macroscopic observations, often correlated with imaging and clinical data, provide indirect evidence of increased ICP. For example, swollen gyri and flattened sulci observed during gross examination can suggest cerebral edema, which contributes to elevated ICP [39].

Mild injuries might only show subtle changes, such as small petechial hemorrhages and minor axonal damage, often requiring specialized staining techniques for detection [40]. In contrast, moderate to severe TBIs typically exhibit more pronounced features, including extensive hemorrhages, significant axonal disruption, and widespread neuronal death. The classification of TBI into focal and diffuse damage further highlights these differences. Focal injuries, such as contusions and lacerations, are characterized by localized damage that can be easily identified during histological examination. In contrast, diffuse injuries, such as DAI, involve widespread damage that may not be immediately apparent without detailed histopathological analysis.

It is crucial to emphasize that while histopathological evaluation provides invaluable insights into the structural and cellular consequences of TBI, the integration of these findings with clinical and imaging data is essential for a comprehensive assessment. This combined approach ensures accurate characterization of the extent and impact of brain injuries following road traffic accidents.

### 3.6. Means of Prevention for Head Trauma

Forensic pathology plays a crucial role in assessing the presence and effectiveness of protective systems, such as helmets and seatbelts, in fatal head trauma cases. Postmortem examinations can provide critical insights into whether such protective measures were used and how they influenced injury patterns.

Helmets, for instance, offer a remarkable reduction in the risk of head, brain, and severe brain injuries by 63 to 88% for bicyclists of all ages [41,42]. A forensic autopsy can reveal various signs indicating whether a helmet was worn at the time of trauma. Patterned abrasions or contusions on the forehead or scalp may correspond to helmet padding or straps. The absence or reduction of skull fractures in expected impact zones suggests that the helmet mitigated impact force. The presence of helmet debris or fibers embedded in wounds may further confirm its use. Additionally, helmets can alter the distribution of force upon impact, leading to diffuse brain injuries rather than localized contusions. Correct helmet use can notably reduce the risk of death in a crash by more than six times, and the risk of brain injury by up to 74% [43].

Seatbelts are equally essential in safeguarding vehicle occupants. Studies have shown that not wearing a seatbelt is significantly associated with more severe head injuries, increased hospital admissions, and a higher likelihood of requiring intensive care or surgery [42]. Seatbelt usage can also be inferred from characteristic forensic findings. The “seatbelt sign”, a linear bruising across the chest and abdomen, is a strong indicator of seatbelt use at the time of trauma. Sternal and clavicular fractures often result from force exerted by a three-point seatbelt. Abdominal injuries, including bowel perforation or mesenteric tears, may suggest lap belt restraint. In contrast, neck hyperflexion injuries, more commonly found in unrestrained passengers, indicate the absence of seatbelt use.

Enhancing vehicle safety features is another critical strategy for reducing the incidence of cerebral hemorrhage and skull fractures in road traffic accidents. Modern vehicles are increasingly being equipped with advanced safety technologies designed to protect occupants during collisions. Features such as anti-lock braking systems (ABS), electronic stability control (ESC), and advanced airbag systems can significantly reduce the severity of injuries sustained in accidents. Forensic examination can assess the impact of such features. Facial fractures and thoracic injuries can indicate airbag deployment and interaction. Severe cerebral trauma with skull fractures may suggest a lack of airbag deployment or ineffective protection. Patterned impact injuries can help to reconstruct the victim’s position and movement inside the vehicle during the crash.

Improvements in road design and maintenance are essential strategies in mitigating the risks associated with road traffic accidents. Simple measures, such as enhancing road markings, installing adequate lighting, and ensuring regular maintenance, can create safer driving conditions. Properly designed roads can reduce the occurrence of accidents by addressing high-risk areas where crashes are more likely to occur. Additionally, incorporating features such as pedestrian crossings, bike lanes, and traffic calming measures can protect vulnerable road users, including pedestrians and cyclists. These preventive strategies are vital in reducing the overall number of accidents and the severity of injuries sustained, particularly head injuries that often result from poor road conditions.

In road traffic fatalities, forensic pathologists collaborate with crash investigators to reconstruct events leading to death. The correlation between injury patterns, vehicle damage, and witness accounts aids in determining whether protective measures were used appropriately and whether their failure contributed to fatal outcomes. Furthermore, forensic evidence of improper safety equipment use may have implications in legal proceedings, such as liability assessments and insurance claims.

### 3.7. The Role of Virtopsy in the Diagnosis of Head Trauma

Virtopsy leverages advanced imaging technologies such as computed tomography (CT) and magnetic resonance imaging (MRI) to provide critical insights into the types of head injuries sustained from RTAs [44]. In living patients, these imaging modalities are primarily used for diagnostic and therapeutic purposes, identifying acute fractures, hemorrhages, and soft tissue injuries that guide immediate medical interventions. In forensic investigations involving deceased individuals, virtopsy focuses on postmortem documentation and injury characterization, making it a valuable alternative or complement to traditional autopsy methods.

This technique can accurately identify various forms of craniofacial injuries, including comminuted fractures, which are complex breaks involving multiple bone fragments [45]. For instance, multi-slice computed tomography (MSCT) has revealed extensively comminuted fractures of the posterior part and the base of the skull in several cases, highlighting the precision of this method in diagnosing severe head trauma [45]. Moreover, the ability to detect such intricate injuries without the need for a traditional scalpel-based autopsy makes virtopsy a crucial tool in forensic investigations. One of the significant advantages of virtopsy in assessing trauma severity is its non-invasive nature, which allows for the preservation of the body while still providing detailed internal images [46].

This technique is particularly effective in identifying the extent of fractures, soft tissue injuries, and wound severity, offering a comprehensive overview of the trauma endured by the victim. Additionally, postmortem use of CT and MRI can detect a wide spectrum of injuries, including craniofacial, cerebral, thoracic, and osseous injuries [47]. These postmortem imaging techniques allow forensic pathologists to reconstruct the dynamics of the trauma and provide precise documentation of injuries. Furthermore, they enable 3D reconstruction and detailed analysis of parenchymal lesions [48].

The successful implementation of virtopsy in real-world cases underscores its effectiveness and reliability. For example, a case study involving a cyclist who was struck by a motor vehicle demonstrated the utility of postmortem CT in identifying the full extent of injuries sustained during the accident [49]. The cyclist, who was not wearing a protective helmet, suffered significant trauma that was meticulously documented using virtopsy techniques [49]. Postmortem CT revealed critical musculoskeletal injuries, as well as fractures and hemorrhages.

Virtopsy stands out primarily due to its non-invasive nature, which significantly preserves the integrity of evidence [50]. Traditional autopsies often involve extensive dissection. This method ensures that no physical alterations are made to the body, which is particularly beneficial in sensitive forensic cases. For example, virtopsy excels in analyzing complex anatomical regions such as the facial district and the cervical spine, where large incisions would otherwise be required during a conventional autopsy. However, it is necessary to consider that postmortem radiological investigations also have limitations, related, for example, to the visualization of details that can only be observed at autopsy, such as areas of infiltration of the galea capitis. The literature has also demonstrated potential limitations in the visualization of fracture lines and cerebral hemorrhagic areas in subjects undergoing virtopsy and subsequent autopsy.

### 3.8. Limitations

This study acknowledges several limitations in its methodology and scope. First, the use of the term “head trauma” in the literature search, rather than “traumatic brain injury” (TBI), was intended to capture a broader spectrum of injuries, including extracranial and intracranial trauma. While this approach ensures inclusivity, it may have reduced the specificity of the review concerning TBI-focused research. This limitation underscores the importance of differentiating the various types and severities of head injuries described throughout the manuscript.

Second, the review methodology is descriptive rather than meta-analytical. Although qualitative synthesis provides a comprehensive overview of existing literature, it does not offer the quantitative rigor or statistical evaluation of prevalence and outcomes that a meta-analysis could achieve. The heterogeneity of the studies included—ranging from forensic autopsy data to clinical studies and imaging-based investigations—further highlights the challenge of standardizing findings across different methodologies and definitions.

Finally, while this review primarily focuses on postmortem findings in traumatic brain injury (TBI) cases, certain aspects—such as long-term survival data and clinical rehabilitation—are beyond its scope. Future forensic studies should aim to refine postmortem diagnostic criteria and injury classification, particularly in cases involving high-impact trauma, to enhance the understanding of injury patterns and mechanisms. By recognizing these limitations, this manuscript aims to provide a balanced and transparent perspective on the forensic evaluation of head trauma, encouraging future studies to address these gaps through standardized methodologies.

## 4. Conclusions

Despite the advancement of postmortem diagnostic technologies, autopsy represents a definitive assessment in the diagnosis of head trauma. In fact, this examination allows us to ascertain the topography, extension and lethality of head trauma. The comparison between autopsy, engineering, radiological, and inspection data also allows for an accurate reconstruction of the accident. Protective equipment such as belts, helmets, and airbags remain fundamental for the prevention of fatal injuries.
